# Analysis of lower extremity alignment (LEA) in children with recurrent patellar dislocation by EOS system

**DOI:** 10.3389/fped.2023.1291739

**Published:** 2023-10-25

**Authors:** Mingyuan Miao, Haiqing Cai, Li Zhang, Haoqi Cai

**Affiliations:** Department of Orthopedic Surgery, Shanghai Children’s Medical Center Affiliated to Shanghai Jiaotong University School of Medicine, Shanghai, China

**Keywords:** EOS imaging system, 3D analysis, recurrent patellar dislocation, lower extremity alignment, standing position

## Abstract

**Objectives:**

Recurrent patellar dislocation (RPD) greatly affects active young individuals, necessitating the identification of risk factors for a better understanding of its cause. Previous research has connected RPD to lower limb alignment (LEA) abnormalities, such as increased femoral anteversion, tibial external rotation, knee valgus, and flexion. This study aims to use EOS technology to detect RPD-related LEA anomalies, enabling three-dimensional assessment under load conditions.

**Methods:**

A total of 100 limbs (50 in the RPD group, 50 in the control group) were retrospectively analyzed. In the RPD group, we included limbs with recurrent patellar dislocation, characterized by dislocations occurs at least two times, while healthy limbs served as the control group. We used EOS technology, including 2D and 3D imaging, to measure and compare the following parameters between the two groups in a standing position: Femoral neck shaft angle (NSA), Mechanical femoral tibial angle (MFTA), Mechanical lateral distal femoral angle (mLDFA), Medial proximal tibial angle (MPTA), Anatomical femoral anteversion (AFA), External tibial torsion (ETT), and Femorotibial rotation (FTR).

**Results:**

The significant differences between the two groups were shown in NSA 3/2D, MFTA 3/2D, mLDFA 3/2D, MPTA 3D, AFA, FTR. No significant difference was shown in MPTA 2D, ETT between the RPD group and the control group. Further binary logistic regression analysis. Further binary logistic regression analysis was conducted on the risk factors affecting RPD mentioned above. and found four risk factors for binary logistic regression analysis: mLDFA (3D), AFA, NSA(3D), and FTR.

**Conclusions:**

EOS imaging identified abnormal LEA parameters, including NSA, MFTA, mLDFA, MPTA, AFA, and FTR, as risk factors for RPD. Children with these risk factors should receive moderate knee joint protection.

## 1. Introduction

Patellar dislocation (PD) is a common unstable knee disease that affects young individuals (1, 2). It occurs when the patella moves sideways out of trochlea groove of the femoral condyle. The annual incidence rate of pediatric PD has been reported to range from 2/100,000 to 107/100,000, accounting for 0.4% of pediatric emergency hospitalizations in surgical wards (3, 4). This greatly reduces patients’ physical activity and lead to long-term complications in adulthood, such as pain, chronic instability, cartilage deterioration and early-onset osteoarthritis (5, 6). Importantly, patients with prior PD have a 22.7–86.2% risk of experiencing a recurrent patellar dislocation (RPD) (7–9). RPD is an important cause of disability in young and active people. Therefore, correct identification of the risk factors for RPD is crucial for the study of the pathophysiology and etiology of RPD (10, 11).

When the knee joint moves back and forth between flexion and extension, the trajectory of the patella depends on the complex three-dimensional motion sequence between the femur, tibia, and patella. The anatomical basis of these movements is the interaction between the joint surface, bone structure, meniscus, ligaments, and muscles. While the initial onset of patellar dislocation is often attributed to accidental injury, the recurrence of this condition is multifactorial, with contributions from various mechanical factors. These factors include lower extremity alignment (LEA), dysfunction of the medial patellofemoral ligament or internal oblique muscle, excessive tension of the lateral retinaculum, systemic joint relaxation (12–18), as well as the alignment of the knee extension device, namely the Q angle (19, 20), and the shape and depth of the femoral sulcus. Among these factors, LEA is extremely important in the pathophysiology and etiology of RPD (21–24). During complete knee flexion, as the knee transitions from an extended state to a flexed state, the patella moves distally relative to the femur, ultimately aligning with the femoral groove. This movement is guided by the complex rotation of the tibia relative to the femur, which in turn is controlled by the consistency of the femur tibia joint surface and the forces of the muscles and ligaments, with significant individual differences. Previous studies have shown that LEA abnormalities, such as increased femoral anteversion angle, increased tibial external rotation, increased tibial external rotation, knee valgus, and knee reflection, are associated with RPD (21–30).

Several studies have shown that the kinematics of the patellofemoral joint observed in the non-weight-bearing posture are significantly different from the kinematics of the patellofemoral joint observed in the weight-bearing posture (31–33). Compared to non-weight-bearing measurements, weight-bearing conditions can alter the measurement of relative force lines due to changes in gravity, muscle strength, and knee joint geometry. Alfredsson et al. reported a radiographic system for examining and evaluating weight-bearing knee joints, including a model for measuring femoral and tibial rotation, patellar translation, and Q-angle (34–37). These previous studies on LEA for RPD were based on two-dimensional (2D) measurements, such as transverse images from plain films or computed tomography (CT) scans. Due to the influence of radiation source position and limb positioning on 2D measurements, there may be limitations in accurately evaluating these force factors. Therefore, the lower limb function in three-dimensional (3D) evaluation should lead to more clinically relevant conclusions.

We urgently needed a new option that can three-dimensionally evaluate lower extremity alignment under weight-bearing conditions. Fortunately, in 2019, our hospital obtained the EOS-3D imaging system (EOS Imaging, Paris, France), which has been applied in many centers around the world. The system can scan the coronal plane and sagittal plane at the same time, and provide the three-dimensional shape of both lower limbs in an upright state under load (38). The two advantages of this system are that it exposes patients to lower radiation doses than traditional radiography (39–41). In addition, traditional radiography uses point source geometry, which can lead to spatial distortion. The EOS system uses a collimator to generate parallel beams, thus minimizing the aforementioned spatial distortion (38).

The purpose of this study was to use EOS technology to identify LEA anomalies related to RPD, which allows for a three-dimensional evaluation of LEA under load conditions.

## 2. Materials and methods

### 2.1. Patient selection

#### 2.1.1. Inclusion criteria

(1) Patients with recurrent patellar instability, having experienced at least two patellar dislocations, despite non-operative treatment. (2) History of two or more positive imaging signs, including patellar dislocation, fracture of the medial patellar medial facet or the lateral femoral condyle, or injuries to the vastus medialis obliquus and the medial patellofemoral ligament on radiographs or magnetic resonance imaging (15, 42). (3) Both lower limbs were analysed to determine whether both knees showed symptoms of RPD. RPD diagnosis is performed by Dr. Haiqing Cai, a paediatric knee disease specialist with 25 years of experience.

#### 2.1.2. Exclusion criteria

(1) Patients with a single episode of patella dislocation. (2) Patients with a history of prior knee surgery. (3) Patients with a history of other knee injuries or dislocations due to direct trauma. (4) Patients with other types of patella dislocation, including habitual or fixed dislocation. After capturing EOS images, we conducted visual assessments to detect limb motion, excluding any images displaying S-shaped curves or bone cortex discontinuity as they were deemed unacceptable.

#### 2.1.3. Control group

Healthy young volunteers without knee complaints or a history of knee injuries were chosen. We reviewed the outpatient medical history system, identified patients who had undergone systemic EOS, and selected individuals who did not exhibit unequal lower limb lengths, abnormal power lines, or discomfort-related complaints for the control group. We tried to select patients around 14 years old who could match the age range of RPD patients. Because these patients just underwent regular EOS during routine follow-up, we only needed to retrieve the data from the database, so there was no need to ask these patients to take EOS again.

### 2.2. EOS examination and analysis

#### 2.2.1. The EOS imaging system

**Figure 1:**
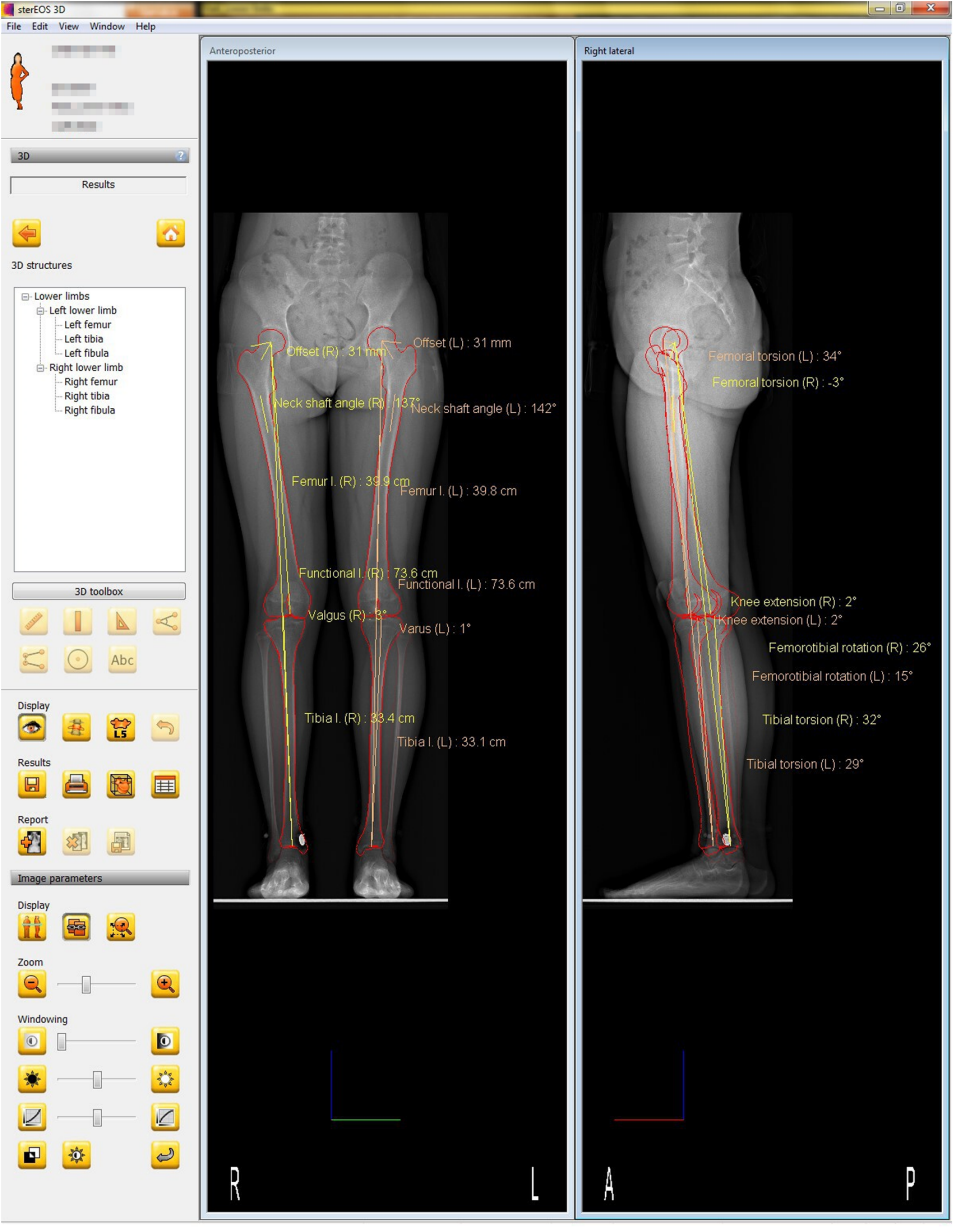
SterEOS workstation (EOS imaging Inc., Paris, France).

Anteroposterior and lateral images (tube voltage: 80 kV; tube current: 200 mA) were acquired simultaneously using the EOS imaging system with the patient in an upright posture to bear the physiological load. The patients applied the load evenly to both lower extremities, with the patella facing forward, and then rotated 15° in both directions to avoid superimposition of the anatomical references on the lateral radiographs for a total of two EOS images. Each 3D model was reconstructed from these biplanar x-ray images using a sterEOS workstation (EOS Imaging Inc., Paris, France). The 3D models of the lower extremities were obtained using semi-automatic adjustments of the anatomical reference points over the bone contours followed by fine manual manipulation (Figure 1) (43–46).

#### 2.2.2. The following variables were measured and analyzed

(1) NSA: Femoral neck shaft angle (NSA)
(2) Mechanical femoral tibial angle (MFTA)

The frontal femoral plane represents the angle between the femoral mechanical axis and the tibial mechanical axis, with a positive angle for valgus and a negative angle for varus alignments.

(3) Mechanical lateral distal femoral angle (mLDFA)

The frontal femoral planet represents the lateral angle between the femoral mechanical axis and the axis across the most distal point of the medial and lateral condyles and is always expressed as a positive angle.

(4) Medial proximal tibial angle (MPTA)

The frontal femoral plane is the medial angle between the tibial mechanical axis and the line connecting the centers of the medial and lateral tibia plateaus.

(5) Anatomical femoral anteversion (AFA)

This represents the angle between the femoral neck axis and the bicondylar axis (the axis between the centers of the spheres fitted to the medial and lateral femoral condyles). It is measured by projecting it onto a plane perpendicular to the mechanical axis of the femur. A negative value indicates femoral retroversion.

(6) External tibial torsion (ETT)

This represents the angle between the line tangential to the posterior portion of the tibial plateau and the bimalleolar axis. It is measured by projecting it onto a plane orthogonal to the mechanical axis of the tibia with a positive angle for external rotation (when the malleoli are turned externally in relation to the tibial plateau) and a negative angle for internal rotation (when the malleoli are turned internally in relation to the tibial plateau).

(7) Femorotibial rotation (FTR)

It is the angle between the posterior bicondylar axis and the axis in contact with the posterior part of the tibial plateau with a positive angle for external rotation of the tibial plateau relative to the femoral condyles and a negative angle when it is internally rotated.

#### 2.2.3. The reference axes were defined as follows

(1) Femoral mechanical axis: the line connecting the center of the femoral head and the center of the trochlea.
(2) Femoral anatomical axis: the line drawn down the center of the femur's diaphysis.
(3) Tibial mechanical axis (correspond with tibial anatomical axis): the line connecting the center of the tibial spines and the center of the distal articular surface of the tibia.

### 2.3. Statistical analysis

The Student's *t*-test without correspondence was used to compare the mean values between the two groups. For these parameters, which had significant and clinically important differences between both groups according to the Student's *t*-test, the relationships between each parameter of the LEA and RPD were assessed using binary logistic regression. All analyses were performed using SPSS version 24.0 (IBM Corp., Armonk, NY), and statistical significance was set at an alpha level of 5%.

## 3. Results

### 3.1. Cohort characteristics

We gathered data from a cohort of 31 patients with recurrent patellar dislocation (RPD), comprising 19 with bilateral involvement and 12 with unilateral symptoms. This allowed us to collect a total of 50 affected limbs and 12 healthy limbs from the RPD group. Additionally, we included 19 patients in the control group, totaling 38 healthy lower limbs. In summary, our study involved a collection of 50 affected limbs and 50 healthy limbs for analysis. Among the limbs affected by RPD, 10 belonged to male patients and 40 belonged to female patients, with an average age of 13.6 ± 1.73793 years. There were 22 on the right side and 28 on the left side. Among the healthy limbs, 20 belonged to males and 30 belonged to females, with an average age of 12.8 ± 2.28571 years. There were 28 on the right and 22 on the left (Table 1).

**Table 1:** Summary of demographic and clinical data of RPD and control groups.

Parameter	RPD group	Control group	*P* value
Age	13.60 ± 1.73	12.80 ± 2.28	0.52
Gender			
(Male/female)	10/40	20/30	0.029
Limbs(right/left)	22/28	28/22	0.23
NSA 3D	130.41 ± 5.35	133.76 ± 6.65	0.007*
NSA 2D	133.26 ± 6.03	136.24 ± 7.84	0.036*
MFTA 3D	2.52 ± 2.53	0.65 ± 3.00	0.001*
MFTA 2D	1.11 ± 2.69	−0.16 ± 3.10	0.031*
mLDFA 3D	83.92 ± 2.67	85.60 ± 2.31	0.001*
mLDFA 2D	84.81 ± 3.18	86.28 ± 2.54	0.012*
MPTA 3D	88.37 ± 2.04	87.21 ± 3.37	0.040*
MPTA 2D	86.93 ± 2.00	86.61 ± 2.89	0.521
AFA	25.96 ± 10.77	17.53 ± 15.76	0.002*
ETT	30.34 ± 7.04	29.75 ± 8.47	0.704
FTR	7.26 ± 8.52	1.95 ± 9.26	0.004*

RPD, recurrent patella dislocation; NSA, femoral neck shaft angle; MFTA, mechanical femoral tibial angle; mLDFA, mechanical lateral distal femoral angle; MPTA, medial proximal tibial angle; AFA, anatomical femoral anteversion; ETT, external tibial torsion; FTR, femorotibial rotation.

* *p* < 0.05.

### 3.2. Lower extremity alignment (LEA)

The mean values and standard deviations of the alignment parameters are described in Table 1. Significant and clinically important differences between the two groups were shown in NSA 3/2D, MFTA 3/2D, mLDFA 3/2D, MPTA 3D, AFA, and FTR. There was no significant difference in MPTA 2D and ETT between the RPD group and the control group, respectively (Table 1).

### 3.3. Logistic regression analysis

Further binary logistic regression analysis was conducted on the risk factors affecting RPD mentioned above. Because there may be a linear relationship between MFTA, mLDFA, or MPTA, it was found that the *P* value of the Hosmer and Lemeshow test of mLDFA was higher (0.892) when one of the three was selected and included in the regression analysis model. When MFTA was selected, the *P* value was (0.866). When MPTA was selected, the *P* value was only (0.356). Therefore, we selected four risk factors for binary logistic regression analysis: 3D mLDFA, AFA, 3D NSA, and FTR (Table 2).

**Table 2:** Logistic regression analysis.

Parameters	B	Sig.	EXP(B)	95% C.I. for EXP(B)	
				Lower	Upper
NSA 3D	−0.093	0.037*	0.912	0.836	0.994
LDFA 3D	−0.24	0.024*	0.786	0.638	0.969
AFA	0.051	0.005*	1.052	1.015	1.091
FTR	0.061	0.042*	1.063	1.002	1.127

NSA, femoral neck shaft angle; mLDFA, mechanical lateral distal femoral angle; AFA, anatomical femoral anteversion; FTR, femorotibial rotation.

* *p* < 0.05.

## 4. Discussion

Recurrent knee pain conditions in children and adolescents, such as patellofemoral pain syndrome (PFPS) (47, 48) and RPD, significantly restrict young individuals, especially young athletes, from returning to regular sports activities. Utilizing imaging techniques to identify patients at a higher mechanical risk for these conditions is crucial. EOS technology offers a low-radiation method for measuring the three-dimensional lower limb force lines while patients bear weight. This approach provides unique advantages over traditional x-rays, CT scans, or MRI.

### 4.1. Coronal plane malformation

Our study demonstrated that coronal plane deformities of the lower limbs in RPD patients, including smaller NSA, smaller mLDFA, and larger MPTA, lead to the exacerbation of genu valgus, potentially affecting the Q angle-a potential risk factor for habitual patellar dislocation.

### 4.2. Horizontal rotation deformities

Previous studies have shown that the measurement results of femoral and tibial torsion obtained through EOS imaging are comparable to those obtained from 2D CT scans (49). It has good consistency in the results of measuring femur, tibia, and femoral tibial torsion based on 3D CT reconstruction technology. In Yan's study, there was no trend of increasing (or decreasing) variability between the two methods, as the mean values of CT and EOS measurements increased. This indicates good consistency between the two methods in measuring femur, tibia, and femur tibia torsion (50).

#### 4.2.1. Anatomical femoral anteversion (AFA)

As we know, the strategies for treating RPD vary from case to case (51–53). Recently, excessive femoral anatomical anteversion angle (AFA) has been found in RPD patients (54–56), and recurrence of patellar dislocation has been found by causing distal femoral internal rotation and incorrect coupling between the patella and the femoral trochlear (54, 57, 58). To address this risk factor, some studies have suggested performing distal femoral circumcision (DDFO) for these patients (59–63). However, there is significant controversy regarding the AFA threshold for DDFO. Nelitz et al. (63) proposed a threshold of AFA >25°, while Weber et al. (64) proposed a lower threshold, suggesting AFA >20°. In addition, it is recommended to combine other factors (such as the “J” sign) with AFA when determining DDFO (65, 66).

However, measuring AFA often requires a CT examination of the lower limbs and a large amount of radiation is often avoided by doctors and patients, which cannot be widely used in clinical practice. At the same time, conducting lower limb CT scans on normal individuals is unethical, which may result in insufficient research data for the relevant control population (63–65). However, EOS technology provides a new method for measuring AFA under the premise of a small amount of radiation. The application of this method can obtain AFA data in the physical examinations of healthy individuals, thus providing assistance for more in-depth research.

**Figure 2:**
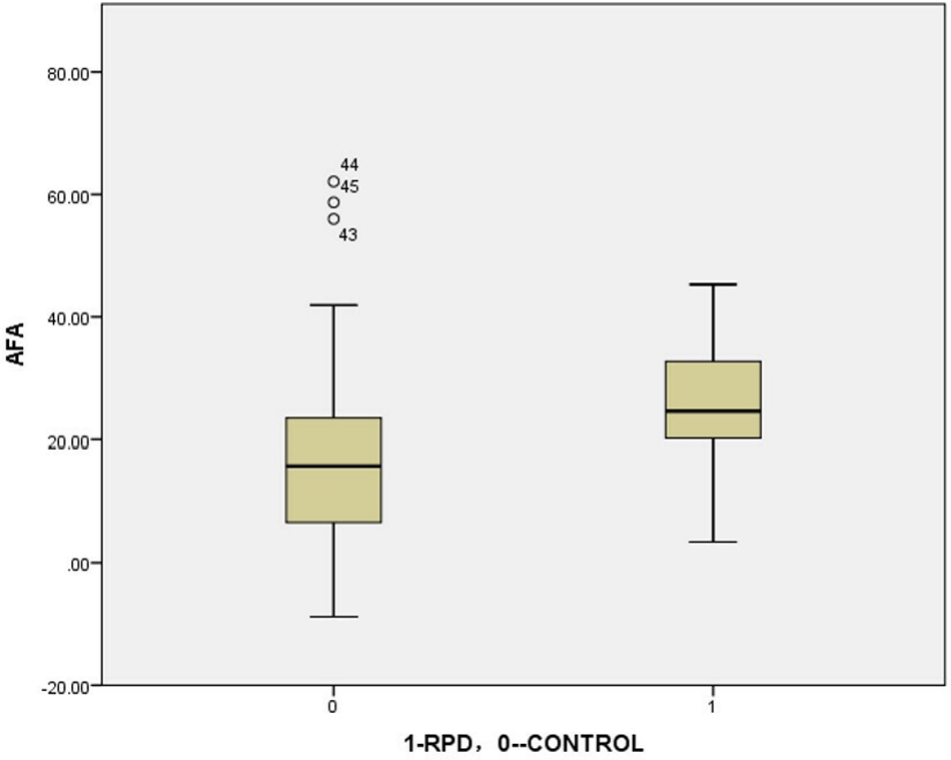
The boxplot of anatomical femoral anteversion: RVD VS CONTROL (analyzed by independent-samples *t*-test)= 23.0°VS 17.5°, *P* = 0.002.

This study applied the latest EOS technology to obtain the AFA when the patient was standing. A similar trend was observed in the femur as in previous studies (with an increase in AFA in RPD compared to normal) (Figure 2 and Table 3) (14, 21, 24, 29, 70). Through statistical analysis, it was found that an abnormal increase in AFA may be one of the risk factors for RPD.

**Table 3:** Summary of the lower extremity alignment measured in the different studies.

Study	Modality	Mean angle RPD VS normal			
		MFTA	AFA	ETT	FTR
Dejour (14)	CT		15.6°VS 10.8°*		
	(NWB)				
Erkocak (22)	CT		14.7°VS 11.6°*	30.2°VS 26.0°*	
	(NWB)				
Takai (29)	CT		30.1°VS 21.7°*	23.5°VS 25.5°	9.1°VS 5.4°*
	(NWB)				
Diederrichs (21)	MRI (NWB)		20.3°VS 13.0°*	25.3°VS 25.3°	9.4°VS 5.7°*
Prakash (24)	CT		19.2°VS 12.0°*	31.4°VS 30.6°	11.5°VS 4.8°*
	(NWB)				
Takagi (66)	CT	1.6°VS 1.0°	30.9°VS 17.0°*	21.6°VS 32.9°*	15.1°VS 5.4°*
	(WB)				
Present study	EOS	3D:	26.0°VS 17.5°*	30.3°VS 29.8°	7.3°VS 2.0°*
	(WB)	2.5°VS 0.7°*;			
		2D:			
		1.1°VS −0.2°*			

MFTA, mechanical femoral tibial angle; ETT, external tibial torsion; AFA, anatomical femoral anteversion; FTR, femorotibial rotation; RPD, recurrent patella dislocation; NWB, non-weight-bearing; WB, weight-bearing.

* *p* < 0.05.

Because all RPD patients in this study had not undergone DDFO surgery, it is not possible to further determine the threshold of DDFO, which requires further research in the future.

#### 4.2.2. External tibial torsion (ETT)

**Figure 3:**
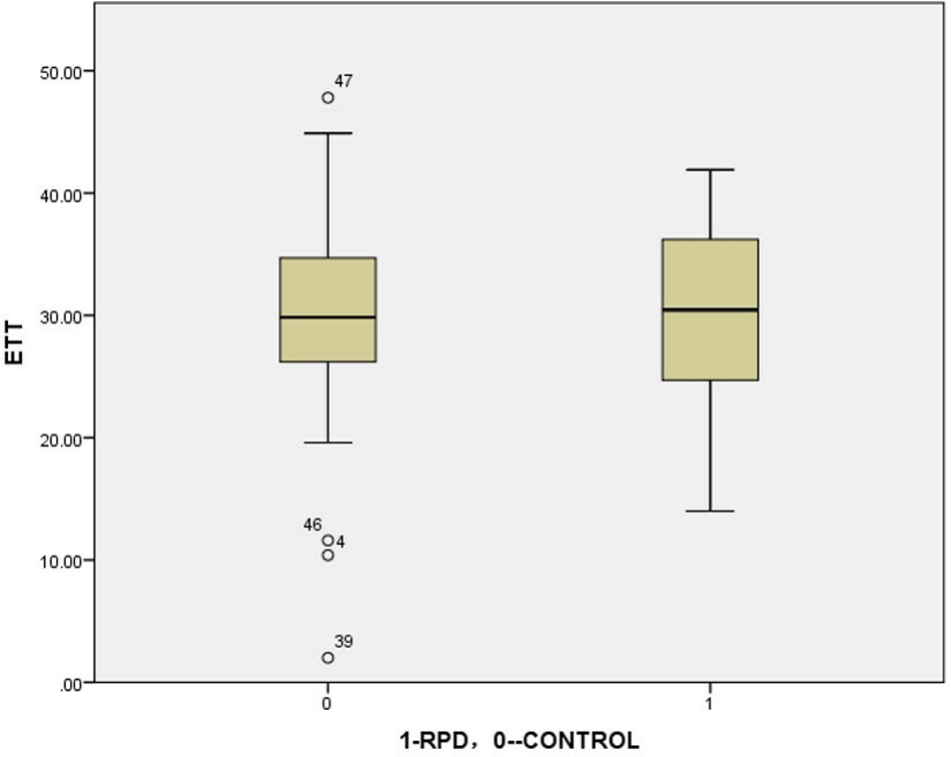
The boxplot of external tibial torsion: RPD VS CONTROL (analyzed by independent-samples *t*-test)= 30.3°VS 29.8°, *P* = 0.704.

In this study, we did not find any abnormal rotation of the tibia in RPD patients relative to the control group (Figure 3), which is consistent with previous studies (Table 3) (21, 22, 24, 29). However, this is different from the research by Takai and Takagi, whose data showed a decrease in tibial torsion in patients with RPD (29, 70).

There is currently little literature on using EOS to measure tibial torsion deformity in patients with RPD. Therefore, due to the lack of relevant comparative data, we can only temporarily understand it as an error caused by different measurement methods. However, from the perspective of RPD pathology, so-called miserable malalignment syndrome may exist, including excessive femoral anteversion, with, or without increased external tibial torsion (71). The position of the tibial tuberosity relative to the femoral trochlea further complicates the process of patella entry into the trochlea (14). The increase in the external rotation of the tibia seems to be more likely to cause external displacement of the tibial tubercle, thereby affecting the trajectory of the patella. This also supports the fact that patients with RPD are more likely to have an increase in tibial external rotation torsion rather than a decrease in tibial external rotation torsion.

#### 4.2.3. Femorotibial rotation (FTR)

**Figure 4:**
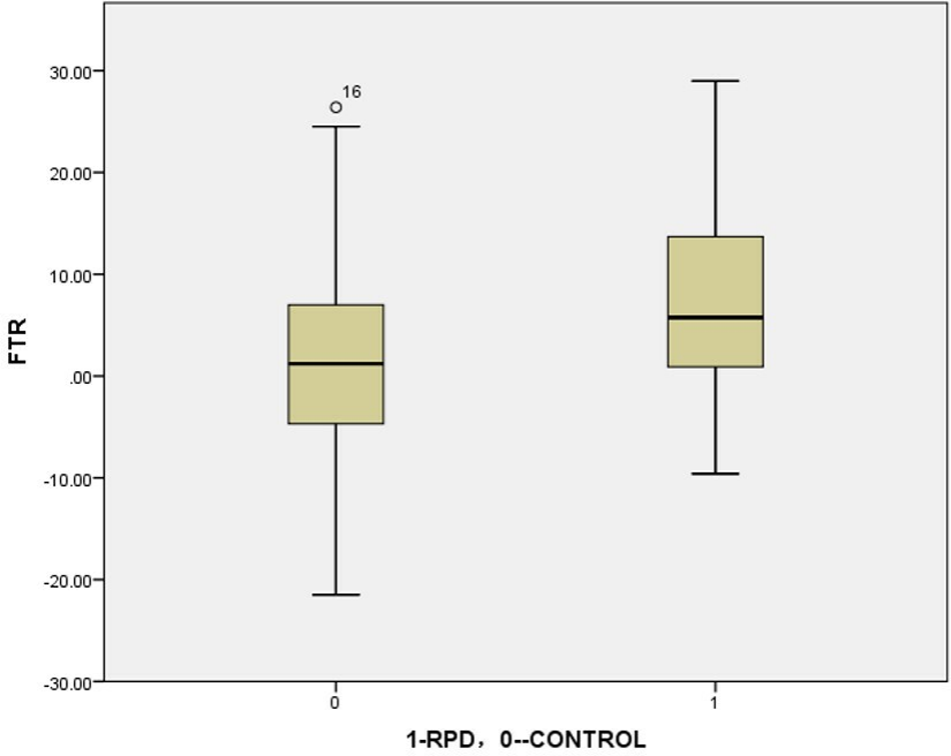
The boxplot of femorotibial rotation: RPD VS CONTROL (analyzed by independent-samples *t*-test) = 7.3°VS 2.0°, *P* = 0.004.

This study showed that, under weight-bearing conditions, in the RPD group, the tibia rotated more outward at the knee joint relative to the femur compared to the control group (Figure 4). This is generally consistent with previous research results (Table 3) (14, 21, 22, 24, 29, 70).

As mentioned above, the position of the tibial tuberosity relative to the femoral trochlea further complicates the process of patella entry into the trochlea (14). Therefore, the lateral rotation of the tibia relative to the femur during the weight-bearing position also affects the position of the tibial tubercle and may result in misclassified malalignment syndrome. Therefore, an excessive increase in FTR may also be one of the risk factors for RPD.

### 4.3. Limitations

This study has several limitations. Firstly, we did not investigate other potential risk factors for RPD, such as foot anomalies. Future research should consider examining a combination of these risk factors. Secondly, due to the higher susceptibility of female patients to RPD, it is essential to increase the number of female patients in control groups for upcoming studies. Additionally, using unaffected limbs from some RPD patients as controls in the control group might potentially impact the final data analysis. Therefore, future research should aim to expand the collection of control group data to reduce this potential source of bias.

## 5. Conclusion

EOS is suitable for LEA screenings in adolescents and children. The 3D-EOS imaging system identified abnormal LEA parameters, including NSA, MFTA, mLDFA, MPTA, AFA, and FTR, as risk factors for RPD. Providing moderate knee joint protection for children with these identified risk factors is advisable to reduce the likelihood of RPD.

## Data Availability

The original contributions presented in the study are included in the article/Supplementary Material, further inquiries can be directed to the corresponding authors.
